# NFAT signalling is a novel target of oncogenic BRAF in metastatic melanoma

**DOI:** 10.1038/sj.bjc.6605277

**Published:** 2009-09-01

**Authors:** R J Flockhart, J L Armstrong, N J Reynolds, P E Lovat

**Affiliations:** 1 Dermatological Sciences, Institute of Cellular Medicine, Newcastle University, Newcastle upon Tyne, UK

**Keywords:** B-RAF, NFAT, melanoma, COX-2

## Abstract

**Background:**

Metastatic melanoma is the most deadly form of skin cancer and with an overall 5-year survival rate of <11%, there is an acute need for novel therapeutic strategies. Activating mutations in the BRAF oncogene are present in 50–70% of cases and contribute to tumourigenesis, thus, defining downstream targets of oncogenic BRAF may help define novel targets for therapeutic intervention. The Ca^2+^/calcineurin-regulated transcription factor, Nuclear factor of activated T-cells (NFAT), is important in the pathogenesis of several human cancers, target genes of which are also known to contribute to melanoma progression. One such NFAT target gene is COX-2, increased expression of which correlates with poor prognosis; however, upstream regulators of COX-2 in melanoma remain undefined. Therefore, the aim of this study was to evaluate NFAT expression and activity in metastatic melanoma and establish whether or not oncogenic BRAF signalling modulates NFAT activity and determine if NFAT is a key upstream regulator of COX-2 in melanoma.

**Methods:**

Nuclear factor of activated T-cells transcriptional activity and protein expression were determined in three human metastatic melanoma cell lines with differing B-RAF mutational status. NFAT activation by oncogenic BRAF^V600E^ was explored by BRAF^V600E^ overexpression and application of the specific MEK inhibitor PD98059. Regulation of COX-2 expression by NFAT was investigated using NFAT-targeted siRNA, calcineurin inhibitors cyclosporin A and FK506, in addition to COX-2 luciferase reporter vectors that selectively lacked NFAT binding sites.

**Results:**

NFAT transcriptional activity was increased in BRAF-mutated melanoma cells compared with wild-type cells. Furthermore, in wild-type cells, overexpression of BRAF^V600E^ increased NFAT activity, which was blocked by the MEK inhibitor PD98059. Using calcineurin inhibitors and siRNA-mediated knockdown of NFAT2 and 4, we show NFAT is required for COX-2 promoter activation and protein induction in metastatic melanoma cells.

**Conclusion:**

NFAT2 and 4 are expressed in human metastatic melanoma cell lines and are activated by oncogenic BRAF^V600E^ via MEK/ERK signalling. NFAT is an important upstream regulator of COX-2 in metastatic melanoma. Furthermore, as the BRAF/MEK/ERK pathway is hyperactive in other malignancies and MEK/ERK are also activated by oncogenic RAS in 30% of all human cancers, the potential to exploit NFAT signalling for therapeutic benefit warrants further investigation.

Malignant melanoma (MM) is the most lethal form of skin cancer and represents an increasing world health problem currently responsible for around 48 000 deaths worldwide each year (www.WHO.int). MM is notoriously unresponsive to current chemotherapy and with an overall 5-year survival rate of <11%, there is an acute need for novel therapeutic strategies (Dhomen and Marais, 2007).

Perhaps the greatest recent advance in understanding risk factors predisposing to melanoma has been the discovery of activating mutations in the *BRAF* gene, occurring in 50–70% of all melanomas (Davies *et al*, 2002; Dhomen and Marais, 2007). *NRAS* mutations are also common in melanoma occurring in up to 30% of cases, and as the occurrence of *NRAS* or *BRAF* mutation in melanoma is mutually exclusive, up to 90% of melanomas harbour a mutated, hyperactive Ras–RAF signalling pathway (Davies *et al*, 2002). Understanding downstream effectors of oncogenic BRAF signalling may, therefore, facilitate the identification of novel therapeutic targets. B-RAF is a serine–threonine kinase belonging to the RAF-extracellular signal-regulated kinase (ERK) kinase (MEK)/ERK pathway, which regulates diverse cellular processes including proliferation, differentiation and apoptosis (Dhomen and Marais, 2007). The *BRAF*^*V600E*^ activating mutation accounts for approximately 90% of *BRAF* mutations in melanoma and BRAF^V600E^ drives melanomagenesis in mice (Dhomen and Marais, 2007; Dankort *et al*, 2009). Inhibition of mutant BRAF signalling in melanoma cell lines reduces proliferation, migration, increases susceptibility to apoptosis induction and ablates tumour formation in mice (Karasarides *et al*, 2004; Sharma *et al*, 2005, 2006). Although *BRAF* is also mutated in up to 80% of benign melanocytic naevi (Pollock *et al*, 2003), the proportion of naevi harbouring a BRAF mutation that progress to melanoma is small, underscoring that further genetic events are required for the development of melanoma. Furthermore, it has now been established that after initial oncogenic activation and proliferation, naevi become senescent and events that overcome or evade senescence are crucial for melanoma development in mice (Michaloglou *et al*, 2005). Nevertheless, the mechanisms by which BRAF signalling promotes melanoma development and/or progression to metastatic disease are not well defined and effective melanoma therapy remains elusive.

Although pre-clinical studies indicate that blocking oncogenic BRAF signalling provides a promising strategy for MM treatment, current clinical trials are yet to demonstrate whether pharmacological targeting of the BRAF/MEK/ERK pathway represents an effective treatment for melanoma as a monotherapy. Although the efficacy of such agents may be improved by combination with conventional chemotherapeutics (Haass *et al*, 2008), the functional redundancy between pathways driving tumour progression and acquired chemoresistance, nevertheless, leaves a demand for alternative drug targets. For example, prolonged treatment of BRAF^V600E^-expressing melanoma cells with the BRAF inhibitor AZ628, leads to the development of clones that maintain high levels of phosphorylated ERK, which continue to proliferate in the presence of drug (Montagut *et al*, 2008). The identification of novel targets for use in combination chemotherapy may therefore help to combat acquired chemoresistance and oncogenic-signalling redundancy.

Nuclear factor of activated T cells (NFAT) proteins were originally defined as a family of Ca^2+^/calcineurin-dependent transcription factors required for T-cell activation (Hogan *et al*, 2003). Outwith the immune system, NFAT is functionally important in the muscle, the bone, the skin (Flockhart *et al*, 2008) and the neural tissue, involved in diverse functions including proliferation, angiogenesis and apoptosis (Hogan *et al*, 2003; Zaichuk *et al*, 2004). Four Ca^2+^/calcineurin-regulated NFAT proteins (NFAT1–4) have been identified that reside hyper-phosphorylated in the cytosol. Dephosphorylation by the Ca^2+^-dependent phosphatase calcineurin evokes NFAT nuclear translocation and transcriptional activation where NFAT drives gene transcription (Hogan *et al*, 2003).

Pertinent to tumour biology, deregulated or abnormal expression of NFAT has been reported in a number of haematological malignancies and solid tumours (Medyouf and Ghysdael, 2008), and we have recently shown that NFAT is preferentially activated by carcinogenic UVB radiation in keratinocytes (Flockhart *et al*, 2008). Furthermore, accumulating evidence suggests that increased NFAT transcriptional activity contributes to both the development and progression of cancer (Buchholz and Ellenrieder, 2007), and identifies NFAT signalling as a potential target for cancer therapy. In the context of melanoma, increased expression of the NFAT target gene cyclooxygenase-2 (COX-2), an inducible enzyme involved in the conversion of arachidonic acid to prostaglandins, correlates with poor prognosis (Kuzbicki *et al*, 2006; Chwirot and Kuzbicki, 2007), suggesting that NFAT signalling may be important in melanoma. However, upstream activators of COX-2 in melanoma and a role for oncogene-driven NFAT activation remain undefined.

Outwith the cancer context, an important role for the Ras/RAF/MEK/ERK signalling axis in regulating NFAT transcriptional activity has been established in immune cells and in myocytes. An effective adaptive immune response relies partly on the production of IL-2 by T cells after T-cell receptor (TCR) activation, which proceeds via MAPK/NFAT signalling. In T cells, dominant negative BRAF or MEK inhibition completely abolished activated TCR-driven ERK activation, IL-2 promoter activation and activation of NFAT1 and 2 indicating the importance of MAPK/NFAT signalling in immune cell biology (Tsukamoto *et al*, 2004). Similarly, stimulating cardiac myocytes with phenylephrine, a ligand that contributes to cardiac hypertrophy, induced NFAT activation that was blocked by MEK/ERK inhibition (Ichida and Finkel, 2001). NFAT therefore serves as a putative downstream target of activated MAPK signalling in non-cancerous cells, however, a role for oncogene-driven MAPK/NFAT signalling has not been described.

The aim of the present study was to test the hypothesis that NFAT may be an important downstream target of oncogenic BRAF in melanoma and that NFAT is involved in regulating COX-2 expression. We show that NFAT is activated by oncogenic BRAF^V600E^ in human metastatic melanoma cell lines via canonical MEK/ERK signalling. In addition, we identify that COX-2 expression is regulated by NFAT in BRAF-mutated melanoma cell lines, therefore highlighting that NFAT can be activated by oncogene mutation and that NFAT regulates downstream factors, which are important in melanoma biology.

## Materials and methods

### Cell culture and chemicals

The human metastatic melanoma cell lines A375, CHL-1 and WM266-4 (American Type Culture Collection, Teddington, UK) were cultured in Dulbecco's modified Eagle's medium (DMEM) (Lonza, Basel, Switzerland) supplemented with 10% foetal bovine serum, penicillin and streptomycin in a humidified incubator at 37°C/5% CO_2_. Primary human melanocytes were identified in cultures of primary keratinocytes obtained from human foreskin samples and isolated by selective trypsinisation, as previously described (Lovat *et al*, 2008). Primary melanocytes were cultured in medium-254 supplemented with melanocyte growth supplements (Invitrogen, Paisley, UK) plus antibiotics. Chemicals purchased were as follows; 12-*O*-tetradecanoylphorbol-13-acetate (TPA), ionomycin, FK506 (Sigma, Poole, UK), Cyclosporin A (Merk Chemicals Ltd, Nottingham, UK) and PD98059 (Promega, Southampton, UK).

### Luciferase assays

Nuclear factor of activated T-cells transcriptional activity was measured using an NFAT-dependent luciferase reporter (p(NFAT)_9_-luc) (Wilkins *et al*, 2004) (a gift from JD Molkentin, Children's Hospital Medical Center, Cincinnati, OH, USA). In all luciferase experiments cells were co-transfected with a renilla luciferase control vector (pRLTK, Promega) and reporter firefly values were normalised to renilla values. Assays were carried out using the dual luciferase assay system (Promega). Cells were seeded in 24-well culture plates and transfected with 0.5 *μ*g of firefly reporter plus 0.0125 *μ*g of renilla control using Lipofectamine 2000 reagent and Opti-MEM medium (Invitrogen) according to manufacturer's instructions. For BRAF overexpression experiments, cells were transfected with 0.5 *μ*g firefly reporter, 0.25 *μ*g BRAF expression vector (pEFmBRAF^WT^ or pEFmBRAF^V600E^ (Davies *et al*, 2002), kind gifts from R. Marais, Institute of Cancer Research, London, UK) and 0.0125 *μ*g renilla. COX-2 luciferase reporters were transfected, as described above, and were gifts from M.A. Iniguez, Centro de Biologia Molecular, Madrid, Spain (Iñiguez *et al*, 2000).

### siRNA-mediated gene silencing

Small interfering RNA (siRNA) targeting NFAT2 (cat. no. SI03057684) and NFAT4 (cat. no. SI03114118) (Qiagen, Crawley, UK) was transfected at a final concentration of 40 nM using Lipofectamine 2000 (Invitrogen) according to the manufacturer's instructions. Cells were incubated for 48 h after transfection then treated with TPA (50 nM) and ionomycin (1 *μ*M) for 4 h and cell lysates were prepared for western blotting.

### mRNA extraction and real-time qPCR

Total mRNA was extracted using an RNeasy kit (Qiagen) and converted to cDNA using a high capacity cDNA reverse transcriptase kit (Applied Biosystems, Warrington, UK) according to the manufacturer's instructions. Expression of NFAT mRNA was determined using a customised gene expression card (Applied Biosystems) and real-time qPCR was carried out using the ABI Prism 7900HT sequence detection system.

### Western blotting

Total protein was extracted from cell pellets, resolved by SDS–PAGE (Web Scientific, Crewe, Cheshire, UK), and transferred to PVDF membranes, blocked and probed as previously described (Flockhart *et al*, 2008). In all, 30 *μ*g of protein was loaded per lane. Antibodies used were as follows: COX-2 (1 : 100), BRAF (1 : 1000), NFAT3 (1 : 1000) and NFAT4 (1 : 500) (Santa Cruz Biotechnology, Autogen Bioclear, Calne, UK), ERK and phospho-ERK (1 : 1000, Cell Signalling Technology, New England Biolabs, Hitchin, UK), and NFAT2 (1 : 1000, BD Biosciences, Oxford, UK). Equal protein loading was confirmed by blotting for *β*-actin (1 : 40 000, Sigma) or GAPDH (1 : 2000, Cell Signalling Technology). Antibody signal was detected using HRP-conjugated secondary antibodies (1 : 2000, Millipore Ltd, Watford, UK) and membranes were visualised using ECL Plus reagent (Amersham Biosciences, Buckinghamshire, UK) and a Fujifilm FLA-3000 fluorescent image analyzer.

### Statistical analysis

Statistical analysis was performed using GraphPad Prism version 5. 00, GraphPad Software (San Diego, CA, USA).

## Results

### NFAT activity and expression in metastatic melanoma cell lines

**Figure 1:**
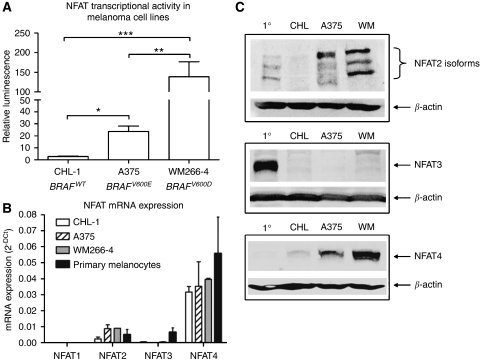
Nuclear factor of activated T-cells (NFAT) transcriptional activity, mRNA expression and protein expression in melanoma cell lines. (**A**) NFAT transcriptional activity measured in three human metastatic melanoma cell lines. BRAF mutational status is indicated for reference. Values are means from four independent experiments carried out in triplicate (± s.e.m.). (**B**) NFAT mRNA expression in primary melanocytes and in three metastatic melanoma cell lines as determined by real-time qPCR. Values are mean 2^−*Δ*Ct^ from RNA derived from two independent cultures (± s.d.). (**C**) Western blots indicate the expression of three previously described NFAT2 isoforms, NFAT3 protein and NFAT4 protein. 1°=primary melanocytes, CHL=CHL-1 cells, WM=WM266-4 cells. Equal protein loading was confirmed by blotting for *β*-actin. Statistical analysis in (**A**) performed by one-way ANOVA, ^*^ *P*⩽0.05, ^**^ *P*⩽0.01, ^***^ *P*⩽0.001.

Basal NFAT transcriptional activity was measured in three human metastatic melanoma cell lines with different BRAF mutational status. CHL-1 cells are BRAF wild-type, whereas A375 and WM266-4 cells harbour *BRAF*^*V600E*^ and *BRAF*^*V600D*^ mutations respectively (Dhomen and Marais, 2007). NFAT transcriptional activity was approximately 10-fold higher in A375 cells and approximately 70-fold higher in WM266-4 cells compared with *BRAF* wild-type CHL-1 cells (Figure 1A), consistent with increased B-RAF activity of BRAF^V600D^ compared with BRAF^V600E^ (Wan *et al*, 2004). At the mRNA level, NFAT2 and to a greater extent NFAT4 were expressed in all the three cell lines and in primary melanocytes, whereas NFAT3 mRNA was expressed exclusively in primary melanocytes, and NFAT1 expression was not detected in any cell type (Figure 1B). We therefore examined the expression of NFAT2, 3 and 4 proteins in primary melanocytes and in the metastatic cell lines. Three previously described NFAT2 isoforms (Hogan *et al*, 2003) were expressed in primary melanocytes but were absent in CHL-1 cells, whereas their expression was higher in A375 cells and had further increased in WM266-4 cells (Figure 1C). NFAT3 protein was exclusively expressed in primary melanocytes (Figure 1C). NFAT4 protein was not detected in primary melanocytes and was expressed at low levels in CHL-1 cells, whereas expression had increased in A375 cells and elevated further in WM266-4 cells (Figure 1C). These data suggest that NFAT2 and NFAT4 are the active NFAT proteins that contribute to the NFAT activity shown in Figure 1A.

### NFAT is activated by oncogenic BRAF^V600E^

**Figure 2:**
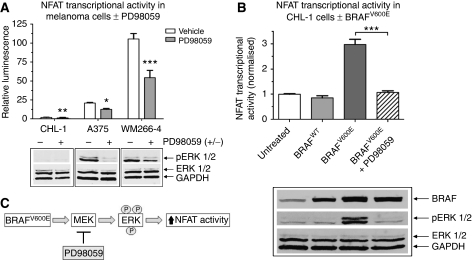
BRAF^V600E^ activates nuclear factor of activated T-cells (NFAT) through a extracellular signal-regulated kinase kinase (MEK)-dependent pathway. (**A**) NFAT transcriptional activity was measured 16 h after treatment with the MEK inhibitor PD98059 (10 *μ*M). Values are means from three independent experiments carried out in triplicate (±s.e.m.). Identically treated samples were prepared for western blotting and membranes were probed for phosphorylated extracellular signal-regulated kinase (ERK) (pERK 1/2) and total ERK (ERK 1/2). (**B**) The effect of BRAF^WT^ or BRAF^V600E^ overexpression on NFAT transcriptional activity in CHL-1 cells was measured by co-transfecting cells with an NFAT luciferase reporter±vectors expressing BRAF^WT^ or BRAF^V600E^. BRAF^V600E^-expressing cells were also treated with PD98059 where indicated. Untreated cells were transfected with NFAT reporter plus transfection reagent only and NFAT luciferase activity was measured 48 h later. Values are normalised to the untreated control and are means from four independent experiments carried out in triplicate (±s.e.m.). Identically treated samples were prepared for western blotting to confirm BRAF^WT^ and BRAF^V600E^ overexpression and downstream ERK phosphorylation. Equal protein loading was confirmed by blotting for GAPDH. Statistical analysis performed by *t*-test (**A**) or one-way ANOVA (**B**), ^**^ *P*⩽0.01, ^***^ *P*⩽0.001 *vs* vehicle control. (**C**) Schematic of BRAF^V600E^-induced NFAT activation.

As data in Figure 1 indicated NFAT transcriptional activity was higher in BRAF-mutated melanoma cells; activation of NFAT by mutant BRAF was further investigated. Inhibition of BRAF/MEK/ERK signalling using the highly specific MEK inhibitor PD98059 resulted in a reduction in NFAT transcriptional activity among all three melanoma cell lines (Figure 2A). PD98059 also reduced the amount of phosphorylated-ERK1/2, thereby confirming inhibition of MEK/ERK signalling and that MEK/ERK signals regulate NFAT activity (Figure 2A). We next assessed the effect of overexpression of BRAF^WT^ or the most common BRAF mutation in melanoma, BRAF^V600E^, on NFAT transcriptional activity in CHL-1 cells. Overexpression of BRAF^WT^ had no effect on NFAT activity, whereas BRAF^V600E^ induced a significant three-fold increase that was blocked by PD98059 (Figure 2B). Western blotting confirmed overexpression of BRAF^WT^ and BRAF^V600E^, BRAF^V600E^-induced ERK phosphorylation and inhibition of BRAF^V600E^-induced ERK phosphorylation by PD98059 (Figure 2B). These data indicate that BRAF^V600E^ increases NFAT transcriptional activity via MEK/ERK-dependent signalling (Figure 2C).

### NFAT regulates COX-2 protein induction in metastatic melanoma cell lines

**Figure 3:**
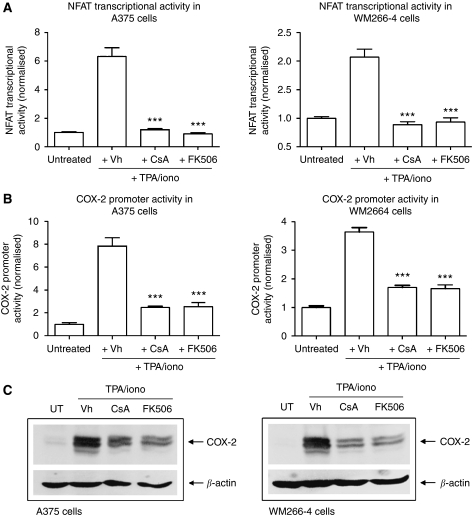
Nuclear factor of activated T-cells (NFAT) inhibition blocks cyclooxygenase-2 (COX-2) protein induction. Cells were transfected with either an NFAT luciferase reporter (**A**) or COX-2 luciferase reporter (**B**) and pretreated for 2 h with vehicle (DMSO, Vh), CsA (1 *μ*M) or FK506 (1 *μ*M) before TPA (50 nM)/ionomycin (iono) (1 *μ*M) treatment. Luciferase activity was measured 4 h after TPA/iono treatment and results are shown for A375 and WM266-4 cells. (**C**) Western blot samples were treated exactly as in luciferase experiments and induction of COX-2 protein is shown for A375 and WM266-4 cells. The three immunoreactive bands present are previously described, glycosylated variants of COX-2 protein. Equal protein loading was confirmed by blotting for *β*-actin. In (**A**) and (**B**) values are means from three independent experiments carried out in triplicate (± s.e.m.). Statistical analysis performed by one-way ANOVA, ^***^ *P*⩽0.001 *vs* cells treated with TPA/iono+Vh.

**Figure 4:**
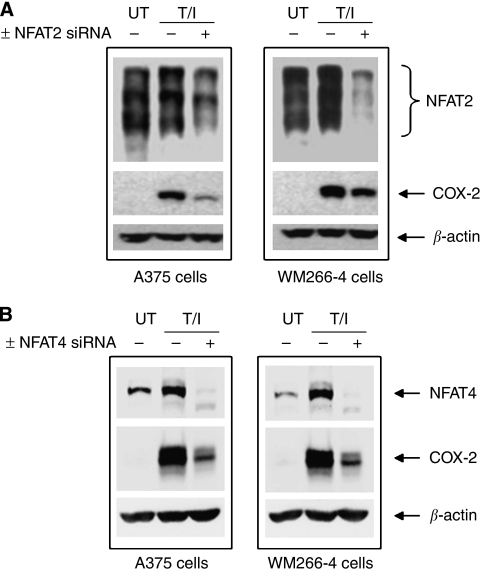
Small interfering RNA (siRNA)-mediated knockdown of nuclear factor of activated T-cells (NFAT) 2 or NFAT4 reduces cyclooxygenase-2 (COX-2) protein induction. WM266-4 and A375 cells were transfected with siRNA targeting either NFAT2 (**A**) or NFAT4 (**B**). 48 h post-transfection, cells were treated with TPA (50 nM) and ionomycin (1 *μ*M) (T/I). UT=untreated control sample. At 4 h after treatment, cell lysates were prepared for western blotting. Membranes were probed with antibodies to detect NFAT2 (**A**), NFAT4 (**B**) and COX-2. Equal protein loading was confirmed by blotting for *β*-actin.

The importance of NFAT signalling in regulating COX-2 promoter activation and protein production was evaluated in BRAF-mutated melanoma cells using COX-2 luciferase reporter vectors that selectively lacked functional NFAT binding sites, calcineurin inhibitors and siRNA-mediated knockdown of NFAT2 and 4. Treating A375 and WM266-4 cells with the classical NFAT activators TPA and ionomycin (TPA/iono) (Hogan *et al*, 2003) increased both NFAT transcriptional activity (Figure 3A) and COX-2 promoter activity (Figure 3B) that was blocked by the calcineurin inhibitors CsA and FK506. This induction/inhibition approach was then employed to establish effects of NFAT inhibition on COX-2 protein induction. Treating A375 and WM266-4 cells with TPA/iono induced a profound increase in COX-2 protein expression that was markedly reduced by CsA and FK506 (Figure 3C). In addition, as NFAT2 and 4 were shown to be expressed in A375 and WM266-4 cells (Figure 1), we decided to ablate NFAT2 and 4 protein using siRNA to show that specific NFAT proteins regulate COX-2 protein induction. Indeed, knockdown of NFAT2 or NFAT4 reduced COX-2 protein induction in these cells (Figure 4).

**Figure 5:**
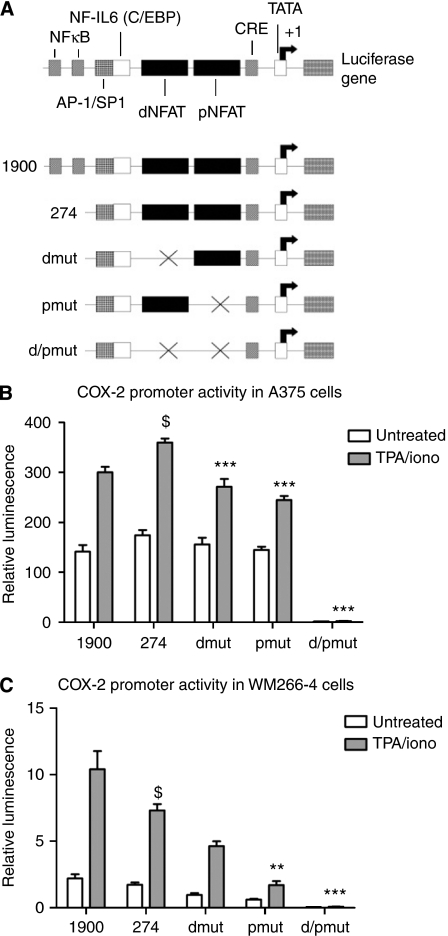
Nuclear factor of activated T-cells (NFAT) regulates cyclooxygenase-2 (COX-2) promoter activity in melanoma cell lines. (**A**) Schematic of the human COX-2 promoter and the COX-2 luciferase reporter vectors used. Cells were transfected with the panel of reporter vectors and treated 24 h later with TPA (50 nM)/ionomycin (iono) (1 *μ*M) for 4 h. Reporter activity is shown for A375 cells (**B**) and WM266-4 cells (**C**). Values are means from three independent experiments performed in triplicate (± s.e.m.). Statistical analysis performed by one-way ANOVA, ^**^P⩽0.01, ^***^ *P*⩽0.001 *vs* control vector containing all functional NFAT binding sites ($).

Additional COX-2 luciferase reporter experiments indicated functional NFAT binding sites in the COX-2 promoter were required for maximal COX-2 induction (Figure 5). The human COX-2 promoter contains two NFAT binding sites and COX-2 luciferase reporter vectors that selectively lacked either one or both of these sites were used to further assess the importance of NFAT as a regulator of the COX-2 promoter (schematic in Figure 5A). Basal COX-2 promoter activity and activity induced by TPA/iono was assessed. Treating A375 and WM266-4 cells with TPA/iono increased the COX-2 promoter activity and this response was reduced in vectors that contained mutated distal (dmut) or proximal (pmut) sites compared with the vector containing both functional NFAT binding sites (274) (Figure 5B, C). Basal promoter activity was slightly reduced in vectors with either dmut or pmut sites, whereas the vector with both NFAT sites mutated reported minimal activity. The reporter vector with both NFAT sites mutated (d/pmut) was not induced at all by TPA/iono in either cell type (Figure 5B, C). Collectively, these data provide evidence that NFAT regulates COX-2 promoter activity and induction of COX-2 protein in metastatic melanoma cells.

## Discussion

Previous studies have shown that NFAT signalling is important in haematological malignancies and solid tumours (Buchholz and Ellenrieder, 2007; Medyouf and Ghysdael, 2008) and that NFAT is activated by environmental carcinogens such as ultraviolet radiation (Flockhart *et al*, 2008), but a role for oncogene mutation-driven NFAT signalling has not been previously described. Although *α*6*β*4 integrin signals stimulate NFAT activity in breast cancer (Jauliac *et al*, 2002), we now identify that NFAT is activated by oncogenic BRAF signalling, which is the most frequently mutated gene in melanoma and a gene frequently mutated in many other cancers. Activating *BRAF* mutations are present in 29–69% of papillary thyroid carcinomas (Wojciechowska and Lewinski, 2006), are also common in colorectal cancers (Ogino *et al*, 2009) and present in 4% of small cell lung cancers (Halilovic and Solit, 2008). Identifying that NFAT functions downstream of mutant BRAF in melanoma, raises the possibility that NFAT may be similarly activated in other BRAF-mutated cancers.

Mechanistically, our data indicate that activation of NFAT by oncogenic BRAF^V600E^ involves canonical BRAF/MEK/ERK signalling. Studies in myocytes and T and B lymphocytes indicate that non-oncogenic Ras/RAF signalling increases NFAT activity via ERK activation concurrent with results from this study (Ichida and Finkel, 2001; Tsukamoto *et al*, 2004). Although MAPK signalling in a non-oncogenic context has been shown to be an important regulator of NFAT activity, the mechanism responsible for this is not completely understood. It is interesting to note that elevated MEK/ERK signalling stimulates AP-1 complex formation which is a well established binding-partner of NFAT that supports NFAT-mediated gene transcription (Macian *et al*, 2000, 2001). In addition, MEK, ERK, calcineurin and NFAT4 are able to form a large molecular weight protein complex in cardiac myocytes, and although active MEK/ERK signalling did not affect NFAT at the nuclear localisation level, MEK/ERK signalling did increase DNA binding of NFAT4 (Sanna *et al*, 2005). In this context, increased NFAT transcriptional activity required AP-1 complex formation (Sanna *et al*, 2005). It is therefore possible that oncogenic Ras/RAF signalling may increase DNA binding and transcriptional activity of NFAT proteins via a MEK/ERK-dependent mechanism that may also require AP-1 complex formation. Further studies to elucidate the mechanism by which MEK/ERK signals increase NFAT transcriptional activity are required particularly, since MEK/ERK/NFAT signalling may be important in other cancers.

Our observation that NFAT activity was higher in V600D-mutated WM266-4 cells compared with V600E-mutated A375 cells, which correlated with the amount of BRAF kinase activity evoked by each mutant is particularly noteworthy. The V600E mutation confers an increase in BRAF kinase activity of approximately 480 times compared with wild-type BRAF, whereas the V600D mutation increases this by approximately 700 times (Wan *et al*, 2004). The level of inhibition of NFAT activity by PD98059 also correlated with the ability of PD98059 to reduce levels of phospho-ERK consistent with the notion that NFAT activity is regulated in-part by MEK/ERK-dependent signalling in melanoma cells. Increased amounts of PD98059 were not used in an effort to further reduce phospho-ERK levels in WM266-4 cells, as increased concentrations of PD98059 can exert non-specific effects. Although MEK inhibition resulted in the downregulation of NFAT activity in BRAF mutant melanoma cells, this effect was also evident in BRAF wild-type CHL-1 cells, although the absolute levels of NFAT activation were substantially lower. This is not surprising as MEK/ERK signalling is still functional, albeit at lower levels in CHL-1 cells. Indeed, the low threshold level of pERK in these cells may promote their sensitisation to MEK inhibition.

The involvement of MEK/ERK activation raises the possibility that other upstream signals activating MEK/ERK may increase NFAT activity. This is highly significant as oncogenic *RAS* (active in approximately 30% of all human cancers) signals partly via MEK/ERK (Schubbert *et al*, 2007), thus further investigation of NFAT signalling in the context of oncogenic Ras/RAF is merited.

The identification of COX-2 as a direct transcriptional target of NFAT in melanoma suggests targeting NFAT/COX-2 signalling may offer a novel therapeutic strategy. COX-2 contributes to tumourigenesis through stimulation of cell proliferation, angiogenesis and tumour metastasis (Buchholz and Ellenrieder, 2007). Recent clinical trials of the specific COX-2 inhibitor celecoxib suggest that COX-2 inhibition may increase the clinical efficacy of temozolomide for melanoma treatment (Gogas *et al*, 2006). Furthermore, as COX-2 is more highly expressed in cutaneous melanoma and metastatic melanoma compared with benign naevi (Chwirot and Kuzbicki, 2007), defining upstream regulators could be valuable for drug development. Pertinent to cancer pathogenesis and metastasis, c-myc and VEGF are additional transcriptional targets of NFAT (Buchholz and Ellenrieder, 2007), further emphasising the relevance of NFAT signalling for melanoma therapy.

Although the classical calcineurin/NFAT inhibitors CsA and FK506 revolutionised transplant biology by inducing immunosuppression via NFAT inactivation in T cells, their potential application in cancer therapy remains untested. The value of CsA and FK506 *per se* may be limited as long term, systemic application in transplant patients causes renal toxicity and increases cancer risk because of reduced immunosurveillance (Botti *et al*, 1998; Sheil, 1998). Novel analogues of CsA and FK506 named ISATX247 and L732531 respectively, exhibit significantly less toxicity while retaining efficacy, and may have a place in future cancer therapy directed at NFAT inhibition (Dumont, 2000; Aspeslet *et al*, 2001; Stalder *et al*, 2003). Alternatively, novel small peptides that block calcineurin/NFAT interaction without inhibiting calcineurin phosphatase activity have exhibited efficacy for inhibiting NFAT *in vivo* in the absence of significant toxicity (Yu *et al*, 2007) and may prove to be valuable as therapeutic NFAT antagonists.

In summary, NFAT is expressed and is transcriptionally active in human metastatic melanoma cell lines and is activated by oncogenic BRAF^V600E^ via canonical MEK/ERK signalling. Although NFAT is overexpressed in other cancers, activation has never previously been linked to the mutation of a specific oncogene. Data indicating that COX-2 expression in melanoma is regulated by NFAT further suggest that NFAT merits additional investigation as a transcription factor important in melanoma biology. Furthermore, given that Ras/RAF signalling is highly activated in many other human cancers, the potential to exploit NFAT signalling for therapeutic benefit clearly warrants further investigation.
